# How Does Adult Children Migration Effects Older Adults Aging in Place? Community and Individual Level Interventions

**DOI:** 10.1093/geroni/igaa057.3229

**Published:** 2020-12-16

**Authors:** Shantha Balaswamy, Rufus Singh

**Affiliations:** 1 The Ohio State University, Columbus, Ohio, United States; 2 Madras School of Social Work, Chennai, Tamil Nadu, India

## Abstract

In India, the increasing emigration by adult children for job opportunity and socio-economic betterment has left vulnerable older adults (OA) to age in place. Almost ‘one of out of ten households in Tamil Nadu state has one or more (e) migrants’ (TN Migration Survey 2015), leaving behind OA who traditionally depend on family as a major source of support. There are few studies on impact of immigration of male adult on wife and children who are left behind, but hardly any research on OA who stay behind. This study aims to understand the social, emotional, economic, and health experiences of OA in one of the districts that has the highest rates of out migration. Data was collected using in-person interviews with 304 randomly selected OA whose adult child had migrated to another country. Nearly half of OA live alone (49.7%), have one or more chronic diseases (60%), require support for meeting their ADLs (90%), and half receive no financial support from adult child (50%) contrary to expectations of providing care for OA in this culture. Findings also reveal that persons over the age of 70 exhibit greater coping skills despite lower functional health, smaller social network, and less financial resources. A significant relationship exists between frequency of visits by adult child and reported levels of anxiety and depression. Implication discussions explore solutions at individual, community and policy levels to address vulnerabilities of the older population who are left behind in villages and towns of India due the globalization related mobility.

